# Actin depolymerisation and crosslinking join forces with myosin II to contract actin coats on fused secretory vesicles

**DOI:** 10.1242/jcs.165571

**Published:** 2015-03-15

**Authors:** Pika Miklavc, Konstantin Ehinger, Ayesha Sultan, Tatiana Felder, Patrick Paul, Kay-Eberhard Gottschalk, Manfred Frick

**Affiliations:** 1 Department of General Physiology, University of Ulm, Albert-Einstein Allee 11, 89081 Ulm, Germany; 2 Institute for Experimental Physics, University of Ulm, Albert-Einstein Allee 11, 89081 Ulm, Germany

**Keywords:** MLCK, ROCK, Cofilin, Lamellar body, Secretion, Surfactant

## Abstract

In many secretory cells actin and myosin are specifically recruited to the surface of secretory granules following their fusion with the plasma membrane. Actomyosin-dependent compression of fused granules is essential to promote active extrusion of cargo. However, little is known about molecular mechanisms regulating actin coat formation and contraction. Here, we provide a detailed kinetic analysis of the molecules regulating actin coat contraction on fused lamellar bodies in primary alveolar type II cells. We demonstrate that ROCK1 and myosin light chain kinase 1 (MLCK1, also known as MYLK) translocate to fused lamellar bodies and activate myosin II on actin coats. However, myosin II activity is not sufficient for efficient actin coat contraction. In addition, cofilin-1 and α-actinin translocate to actin coats. ROCK1-dependent regulated actin depolymerisation by cofilin-1 in cooperation with actin crosslinking by α-actinin is essential for complete coat contraction. In summary, our data suggest a complementary role for regulated actin depolymerisation and crosslinking, and myosin II activity, to contract actin coats and drive secretion.

## INTRODUCTION

Regulated secretion is a fundamental cellular process in many different types of eukaryotic cells. Vesicle contents are released through exocytosis of secretory vesicles. During exocytosis a sequence of highly regulated steps leads to fusion of exocytic vesicles with the plasma membrane, opening of a fusion pore and finally content release (Bean et al., 1994; Lindau and Gomperts, 1991; Rettig and Neher, 2002; Südhof, 2004).

It has been known for decades that actin remodelling plays a role in multiple steps of exocytosis. In particular, actin has been proposed to regulate exocytosis of secretory vesicles during the pre-fusion phase (Nightingale et al., 2012; Porat-Shliom et al., 2013), mainly adjusting the number of vesicles that fuse with the plasma membrane. The actin cytoskeleton provides tracks for trafficking of secretory granules towards the fusion sites (Rojo Pulido et al., 2011; Rudolf et al., 2003), provides a scaffold for anchoring vesicles in close proximity to the plasma membrane (Abu-Hamdah et al., 2006; Gotow et al., 1991; Miyamoto, 1995) and forms a passive barrier to prevent the (premature) fusion of secretory granules with the plasma membrane (Brown et al., 2011; Giner et al., 2005; Orci et al., 1972).

In recent years, however, more and more evidence has arisen showing that actin and the actomyosin complex also regulate secretory output during the so-called exocytic post-fusion phase. It is now well established that actin and myosin are specifically recruited to the surface of the vesicles following fusion with the plasma membrane in various secretory cells. Actin and myosin II coating of fused granules had already been observed several decades ago (Segawa and Yamashina, 1989; Tsilibary and Williams, 1983), but the precise roles for these coats have only begun to emerge in recent years (Nightingale et al., 2012). Actin has been shown to regulate the opening and closure of the fusion pore (Chan et al., 2010; Larina et al., 2007), to stabilise the limiting membranes of fused secretory granules to facilitate content release (Nemoto et al., 2004; Sokac et al., 2003) and, more actively, to provide the force necessary to expel bulky vesicle cargo from fused granules (Jerdeva et al., 2005; Masedunskas et al., 2011; Miklavc et al., 2009; Nemoto et al., 2004; Nightingale et al., 2011; Sokac and Bement, 2006).

Despite the well-established importance of the actomyosin complex for regulating secretion, little is known about the molecular mechanisms that regulate actin coat formation and drive coat contraction on fused granules. In *Xenopus* oocytes specificity for selective coating of fused granules is achieved by membrane-fusion-dependent compartment mixing (Yu and Bement, 2007a). Upon fusion, key components of the plasma membrane can diffuse into the fused secretory granule membrane and act as trigger for local actin assembly [so-called ‘kiss-and-coat’ (Sokac and Bement, 2006)]. Depending on the cell type, Arp2/3 (Gasman et al., 2004; Yu and Bement, 2007a) and formins (Miklavc et al., 2012) have been shown to play a role in actin nucleation; however, given the observed dynamics of actin coat formation it remains possible that an unidentified rapid nucleating system is yet to be discovered (Nightingale et al., 2012). Even less information is available on the mechanisms that drive coat contraction. So far, a role for myosin II in actin coat contraction has been reported in most systems (Jerdeva et al., 2005; Masedunskas et al., 2011; Miklavc et al., 2012; Nemoto et al., 2004; Nightingale et al., 2011; Yu and Bement, 2007b). However, the precise kinetics of myosin II recruitment relative to actin assembly have yet to be determined. Moreover, in several systems, inhibition of myosin II activity does not completely block actin coat contraction, but rather delays it (Masedunskas et al., 2011; Miklavc et al., 2012; Yu and Bement, 2007b). This implies that myosin II is not essential for actin coat contraction, but seems to have a facilitating function, and alternative mechanisms must contribute to effective coat contraction and granule compression. It has been speculated that actin polymerisation alone might be sufficient to compress the exocytic vesicle (Giardini et al., 2003; Sokac et al., 2003). Recent models of cytokinetic actin ring compression in dividing cells have also suggested that the generation of contractile forces is mediated by actin filament depolarisation and crosslinking (Mendes Pinto et al., 2012; Mseka and Cramer, 2011; Sun et al., 2010).

We have recently reported that lamellar bodies are coated with actin following fusion with the plasma membrane in primary alveolar type II (ATII) pneumocytes (Miklavc et al., 2009). Lamellar bodies are large secretory organelles for pulmonary surfactant, a poorly soluble, lipoprotein-like substance responsible for reducing surface tension in lung alveoli. Efficient secretion (expulsion) of surfactant depends on actin coat contraction and vesicle compression (Miklavc et al., 2012). Myosin II is involved in actin coat compression but detailed mechanisms of myosin II activation and coat contraction were still missing.

Within this study we now provide a detailed kinetic analysis of the molecules regulating actin coat contraction of fused secretory granules. We demonstrate that ROCK1 and myosin light chain kinase 1 (MLCK1, also known as MYLK) translocate to fused lamellar bodies and activate myosin II which is recruited to fused lamellar bodies only after actin coat formation. In addition, we provide evidence that ROCK1 also modulates the activity of the actin-severing protein cofilin-1. Moderate cofilin-1 activity and translocation of the actin crosslinker α-actinin are essential for full contraction of the actin coat, likely resulting in effective, force-producing interactions between cytoskeletal elements. In summary, our data support a model in which actin depolymerisation and crosslinking join forces with myosin II to contract actin coats around fused secretory vesicles to drive secretion.

## RESULTS

### Myosin II recruitment to fused lamellar bodies following actin coat formation

**Fig. 1.**
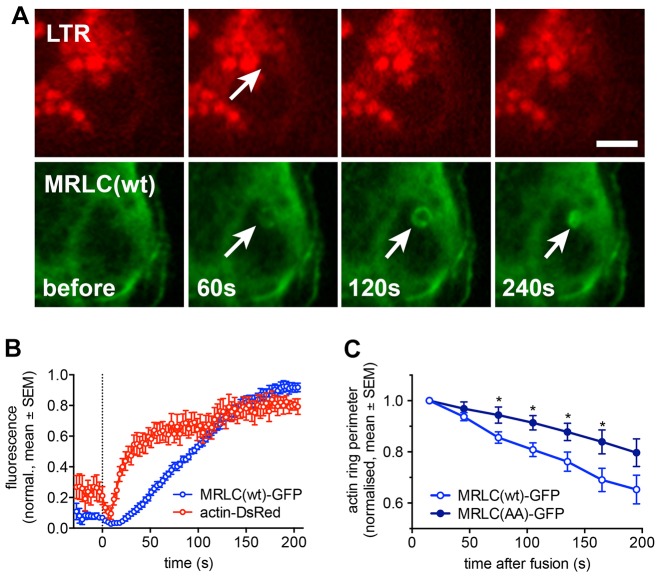
**Myosin II is recruited to fused lamellar bodies following actin coat formation.** (A) Simultaneous imaging of LTR (red) and MRLC–GFP (green) revealed recruitment of MRLC to lamellar bodies upon fusion with the plasma membrane. Lamellar body fusion with the plasma membrane is indicated by the selective decrease in LTR fluorescence due to diffusion of the LTR from the vesicle lumen (arrow, upper row). Time indicates time after fusion. Scale bar: 5 µm. (B) Time course of actin–dsRed (red) and MRLC(wt)–GFP (blue) fluorescence analysed in a circular region of interest around fusing lamellar body. Dashed line denotes time of fusion. Data represent mean±s.e.m. from eight individual fusions. (C) Expression of the non-phosphorylated MRLC mimic [MRLC(AA)–GFP] slowed down actin coat contraction significantly compared to expression of MRLC(wt)–GFP (*P<0.05 for 75–165 s; *n* = 15 and 11 for wt and AA, respectively).

We have recently demonstrated that actin coating and compression of fused lamellar bodies are essential for efficient surfactant secretion. We have shown that myosin II facilitates actin coat contraction, however, precise kinetics of myosin II recruitment were still elusive (Miklavc et al., 2012). To investigate the kinetics of myosin translocation to lamellar bodies following fusion we analysed the translocation of GFP-tagged myosin regulatory light chain (MRLC–GFP, MRLC is also known as MYL2) to lamellar bodies following fusion (Fig. 1A). GFP-tagged wild-type MRLC [MRLC(wt)–GFP] translocation lagged lamellar body fusion by 15.5±1.2 s (*n* = 20, mean±s.e.m.). This was significantly longer than the time it took for actin coat formation (7.4±0.4 s, *n* = 25, *P* = 0.0001) and indicates that myosin II has been recruited to already existing actin coats and is therefore not essential for actin coat formation (Fig. 1B; Fig. 7). Mutants mimicking phosphorylated [MRLC(DD)–GFP] and non-phosphorylated [MRLC(AA)–GFP] MRLC were also recruited to fused lamellar bodies (supplementary material Fig. S1A), suggesting that phosphorylation of MRLC is not solely responsible for recruitment of myosin II to actin coats. However, analysing the effect of MRLC phosphorylation on actin coat compression revealed that actin coat contraction was significantly reduced in cells expressing MRLC(AA)–GFP compared to cells expressing MRLC(wt)–GFP (Fig. 1C; *P* = 0.01–0.03 between 75 s and 165 s). The effect of MRLC(AA)–GFP on coat contraction was similar to the previously observed partial inhibition of coat contraction following pharmacological inhibition of myosin II with (−)-blebbistatin (Miklavc et al., 2012).

### Complementary role for ROCK1 and MLCK1 in regulation of coat contraction

**Fig. 2.**
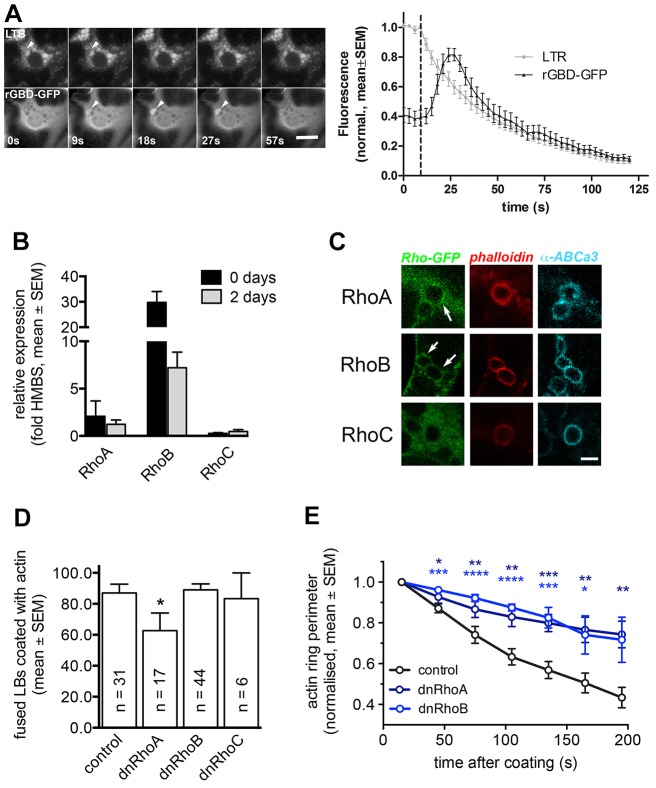
**RhoA and RhoB translocate to fused lamellar bodies and probably regulate coat formation and contraction.** (A) Left: The marker for active Rho GTPases, rGBD–GFP, transiently translocated to the fusing lamellar body (arrowhead, bottom row). Time of fusion was detected by LysoTracker fluorescence decrease (arrowhead, upper row). Scale bar: 10 µm. Right: LysoTracker fluorescence change (indicating vesicle fusion) and rGBD–GFP fluorescence change were measured in a circular region of interest around the fusing lamellar body (*n* = 17). (B) Real-time RT-PCR analysis of RhoA, RhoB and RhoC transcripts in freshly isolated rat ATII cells and ATII cells kept in culture for 2 days. Data are expressed as fold expression compared to the housekeeping gene *Hmbs*. Values are means from three individual cell isolations and are represented as mean±s.e.m. (C) Expression of Rho GTPase isoforms tagged with GFP revealed that only RhoA and RhoB (arrows) but not RhoC translocated to fused lamellar bodies following fusion with the plasma membrane. Fused lamellar bodies can be identified by the actin coat (phalloidin) on the lamellar body membrane (ABCa3). Scale bar: 2 µm. (D) Actin coating of fused lamellar bodies was significantly reduced in cells expressing dnRhoA-IRES–YFP, but not in cells expressing dnRhoB-IRES–YFP or dnRhoC-IRES–YFP. *n* represents number of cells analysed for each condition. Data are represented as means±s.e.m. (E) Expression of dnRhoA-IRES–YFP or dnRhoB-IRES–YFP in cells transfected with actin–dsRED resulted in a significantly decreased coat contraction compared to control cells (*n* = 19, 7 and 7 fusions for control, dnRhoA-IRES–YFP and dnRhoB-IRES–YFP, respectively). *P<0.05; **P<0.01; ***P<0.001; ****P<0.0001.

To further elucidate the regulation of actin coat contraction we next investigated possible mechanisms regulating myosin II phosphorylation on fused lamellar bodies. More than a dozen kinases have been reported to phosphorylate MRLCs of non-muscle myosin II, including myosin light chain kinase (MLCK) and Rho-associated, coiled coil-containing kinase (ROCK) family proteins (Vicente-Manzanares et al., 2009). ROCK can be activated by small GTPases of the Rho family (Riento and Ridley, 2003), which are also involved in actin coat formation in ATII cells (Miklavc et al., 2012). We found that rGBD–GFP, a reporter for active RhoA, RhoB and RhoC, transiently translocated to the vesicle membrane after fusion with the plasma membrane, indicating that there is a rapid activation of Rho signalling pathways on fused lamellar bodies (Fig. 2A). To further elucidate which specific Rho GTPase is responsible for actin coat formation and contraction, we analysed the expression and recruitment of Rho isoforms to fused lamellar bodies. Semi-quantitative RT-PCR revealed that RhoB was by far the highest expressed isoform, that there was substantial expression of RhoA, but hardly any expression of RhoC when compared to housekeeping gene *Hmbs* (Fig. 2B). Moreover, only RhoA–GFP and RhoB–GFP translocated to lamellar bodies upon fusion with the plasma membrane, but not RhoC–GFP (Fig. 2C). These data suggest that that RhoA and/or RhoB, rather than RhoC, are involved in actin coat formation and contraction. To further dissect the role of Rho isoforms for contraction, we expressed dominant-negative (dn) isoforms of RhoA, RhoB and RhoC [RhoA(T19N), RhoB(T19N), RhoC(T19N)] using an internal ribosome entry site (IRES) expression system (pIRES-YFP) to identify transfected cells. Only expression of dnRhoA (62.6%±11.4, *n* = 17, mean±s.e.m.), but not RhoB (89.05%±11.4, *n* = 44) or RhoC (83.3%±16.7, *n* = 6) significantly (*P* = 0.03) reduced actin coat formation on fused lamellar bodies when compared to control cells (87.0%±5.6, *n* = 31) (Fig. 2D), suggesting that RhoA is the predominant isoform in inducing coat formation. However, when analysing actin coat contraction, both dnRhoA and dnRhoB significantly slowed down actin coat contraction (Fig. 2E). Both isoforms have been shown to signal to ROCK1 and MLCK (Ridley, 2006) and hence it is possible that either of the two isoforms contributes to regulating coat contraction.

**Fig. 3.**
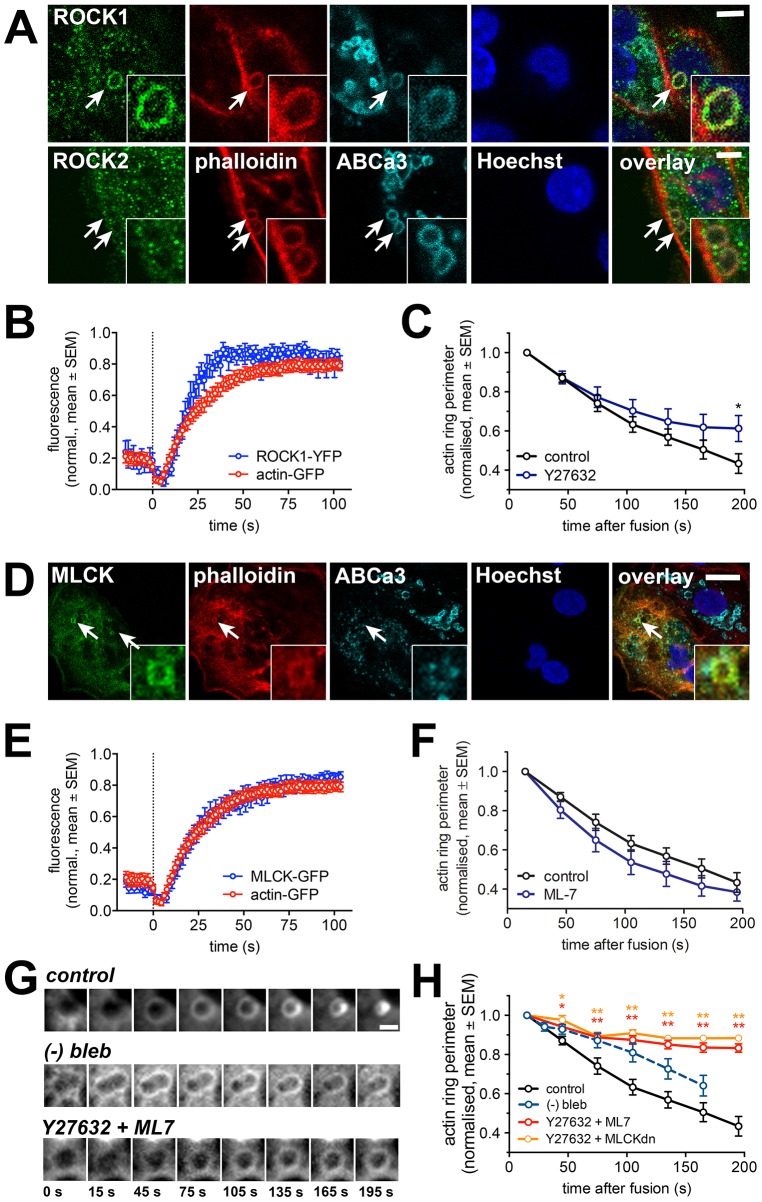
**ROCK1 and MLCK influence actin coat compression.** (A) Immunostaining with anti-ROCK antibodies showed that ROCK1 but not ROCK2 was present on actin coats around fused vesicles. Phalloidin was used to detect actin coats, anti-ABCa3 antibody to mark the lamellar body membrane and Hoechst 33342 dye to mark the nucleus. Scale bar: 5 µm. Inserts show enlarged view of the vesicles. (B) The kinetics of ROCK1–YFP recruitment to fused lamellar bodies closely resembled the accumulation of actin–GFP at the actin coat. Fluorescence intensity was measured at the circular region of interest on lamellar bodies at the time of fusion (dotted line; *n* = 22 and 6 fusions for actin–GFP and Rock1–YFP, respectively). (C) Inhibition of ROCK by Y27632 (10 µM) on actin–GFP-transfected cells showed similar initial vesicle compression as control, however the compression was significantly reduced at time = 195 s after coat formation (*P* = 0.03; *n* = 19 and 13 for control and Y27632, respectively). (D) MLCK–GFP colocalised to phalloidin-stained actin coats on the lamellar body membrane (stained with anti-ABCa3 antibody) in immunostained ATII cells. Scale bar: 10 µm. (E) MLCK–GFP fluorescence change at fusing lamellar bodies compared to fluorescence change in actin-GFP. The time of fusion (measured by LTR fluorescence decrease) is indicated by a dashed line (*n* = 22 and 14 fusions for actin and MLCK, respectively). (F) Pharmacological inhibition of MLCK with ML7 (30 µM) did not significantly change the compression rate of actin coats compared to control in actin–GFP-transfected cells (*n* = 19 and 16 fusions for control and ML7, respectively). (G) Image sequence of actin coat compression in untreated cells (control) and cells treated with either (−)-blebbistatin or Y27632 + ML7. Images depict actin-GFP fluorescence. Time = 0 indicates last image frame before fusion. Scale bar: 2 µm. (H) Simultaneous inhibition of ROCK and MLCK with either combined inhibition by Y27632 and ML7 or application of Y27632 on cells transfected with dn MLCK resulted in significant inhibition of vesicle compression rate (*n* = 19, 19 and 3 for control, Y27632+ML7 and Y27632+dn MLCK respectively). This effect was more prominent than inhibition of myosin II activity by (−)-blebbistatin (dashed line) (Miklavc et al., 2012). *P<0.05, **P<0.01.

In addition to directly phosphorylating MRLC, ROCK also inhibits myosin light chain phosphatase activity which further potentiates myosin activation (Vicente-Manzanares et al., 2009). Semi-quantitative RT-PCR revealed that ROCK and MLCK were expressed in primary ATII cells; ROCK isoforms 1 and 2 where expressed almost equally, but only MLCK1, and not MLCK2 and MLCK3, expression could be detected (supplementary material Fig S1B). Immunofluorescence staining further revealed that ROCK1 was recruited to actin-coated lamellar bodies, whereas we did not detect ROCK2 on lamellar bodies (Fig. 3A). In live-cell experiments ROCK1–YFP translocated to fused lamellar bodies with a delay of 7.0±0.8 s (*n* = 6), similar to the delay observed for the initiation of actin coat formation and significantly before MRLC translocation (*P* = 0.001) (Fig. 3B; Fig. 7). Similarly, MLCK1–GFP translocated to fused lamellar bodies at the time of actin coat formation (6.0±0.5 s following fusion, *n* = 20) and significantly before translocation of MRLC (*P* = 0.0001) (Fig. 3D,E; Fig. 7).

Surprisingly, neither inhibition of ROCK1 with 10 µM Y27632, nor inhibition of MLCK with 30 µM ML-7 had any significant effect on the initial contraction of the actin coat (Fig. 3C,F; Fig. 6; *n* = 13, 19, and 16 for Y27632, control and ML7, respectively). Whereas ML-7 did not show any significant effect on actin coat contraction, treatment with Y27632 significantly (*P* = 0.03) inhibited the late stages of actin coat contraction (>190 s after fusion) (Fig. 3C). This was also reflected in significantly reduced compression rate after Y27632 treatment at 135 s and 195 s after fusion (*P* = 0.02) compared to control (Fig. 6). Ultimately, inhibition of ROCK significantly inhibited the completion of actin coat contraction, with only 15.2±2.6% (*n* = 107) coats fully contracting within 10 min compared to 65±4.1% (*n* = 94) under control conditions (*P*<0.0001, supplementary material Fig. S2A,B).

However, combined inhibition of ROCK and MLCK resulted in almost complete inhibition of coat contraction from the start and led to a highly significant reduction in the rate of actin coat compression at any stage following fusion (*n* = 18, Fig. 3G,H; Fig. 6). Under these conditions no compression of the fused vesicle can be observed, as revealed by simultaneous imaging of actin coats and lamellar body membranes following fusion with the plasma membrane (supplementary material Fig. S2C,D). The same effect was observed when ROCK was inhibited (Y27632) in cells overexpressing a dominant negative mutant of MLCK (*n* = 3, Fig. 3H). The combined effect of ROCK and MLCK inhibition on actin coat contraction was also stronger than the effect of myosin II inhibitor (−)-blebbistatin (Fig. 3H; Miklavc et al., 2012), suggesting that translocation of ROCK1 and MLCK1 to fused lamellar bodies did not solely induce phosphorylation of MRLC and activation of myosin II to promote actin coat contraction, but could also activate complementary mechanisms for coat contraction.

### Regulating cofilin activity is necessary for efficient coat contraction

**Fig. 4.**
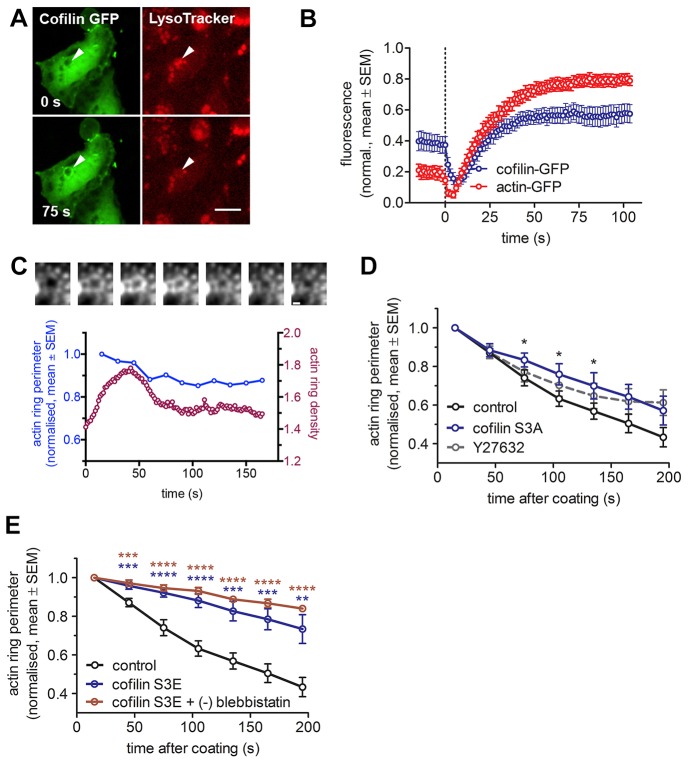
**The role of cofilin in lamellar body compression.** (A) Cofilin–GFP (green) translocated to lamellar bodies after fusion. The time of fusion was assessed by LysoTracker Red (red) diffusion out of the vesicle, resulting in fluorescence decrease (arrowhead, right). Scale bar: 10 µm. (B) Cofilin–GFP translocated to lamellar bodies with a slight delay compared to actin–GFP. Fluorescence change was measured at the circular region of interest around lamellar body at the time of fusion (dotted line) (*n* = 24 and 22 fusions for cofilin and actin, respectively). (C) Inhibition of ROCK with Y27632 resulted in decomposition of actin coat. The image series (top) shows an actin coat around a single fused vesicle in a Y27632-treated cell. Actin coat density decreased without vesicle compressing, which is shown on the graph below. Scale bar: 1 µm. (D) Expression of the constitutively active cofilin S3A–DsRed mutant in actin–GFP-transfected cells resulted in decreased vesicle compression, which was not significantly different from compression in cells treated with Y27632 inhibitor (*n* = 19, 13 and 12 fusions for control, Y27632 and cofilin S3A, respectively). (E) Expression of cofilin S3E–DsRed mutant (dominant negative) in actin–GFP-transfected cells resulted in significantly decreased vesicle compression rate compared to control from the start (*P*<0.01, *n* = 19 and 13 fusions for control and cofilin S3E–DsRed, respectively). Addition of (−)-blebbistatin resulted in further, yet non-significant reduction of the compression rate (*n* = 4). *P<0.05, **P<0.01; ***P<0.001, ****P<0.0001.

We next aimed at identifying the nature of the complementary contraction mechanisms regulated by ROCK1 and MLCK1. It has been reported that active ROCK, in addition to its effects on MRLC phosphorylation, also leads to phosphorylation and thereby inactivation of the actin-severing protein cofilin (Ridley, 2006; Riento and Ridley, 2003). Hence, we next investigated a potential effect of cofilin activity on actin coat contraction. Initial experiments confirmed that cofilin–GFP translocated to fused lamellar bodies shortly after initiation of actin coat formation (9.0±1.3 s, *n* = 20 compared to 7.4±0.4 s, *n* = 25, respectively, mean±s.e.m.) (Fig. 4A,B; Fig. 7). Following treatment with Y27632, actin coats started to disintegrate before completion of contraction (Fig. 4C) suggesting that inhibition of ROCK1 affects cofilin activity on actin coats resulting in increased severing activity. Moreover, the effects of expressing a constitutively active mutant of cofilin (cofilin S3A) resembled the moderate effect of ROCK inhibition on actin coat contraction (Fig. 4D). In summary, these data suggest that ROCK1 does not solely activate myosin II but also prevents premature actin filament breakdown by cofilin to drive coat contraction. However, such model does still not explain the incomplete effect of myosin II inhibition on vesicle compression.

### Actin depolymerisation and crosslinking is essential for coat contraction

**Fig. 5.**
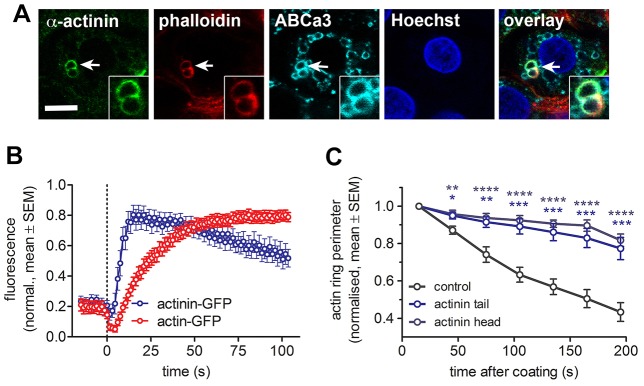
**The role of actin crosslinking protein α-actinin in lamellar body compression.** (A) Immunostaining revealed colocalisation of anti-α-actinin antibody with actin coats (stained by phalloidin) around fused lamellar bodies. Staining for the ABCa3 transporter was used to mark the lamellar body membrane and Hoechst 33342 dye to stain the nucleus. Inserts show enlarged view of the vesicles. Scale bar: 5 µm. (B) α-Actinin–GFP was recruited to fused lamellar bodies at the time of actin coat formation. The fluorescence change was measured at the circular region of interest on lamellar bodies at the time of fusion (dotted line; *n* = 14 and 22 fusions for α-actinin and actin, respectively). (C) Expression of dominant negative α-actinin tail or head domains in actin–GFP-transfected cells resulted in almost complete inhibition of vesicle compression (*n* = 19, 7 and 14 for control, actinin tail and head, respectively). *P<0.05, **P<0.01; ***P<0.001, ****P<0.0001.

**Fig. 6.**
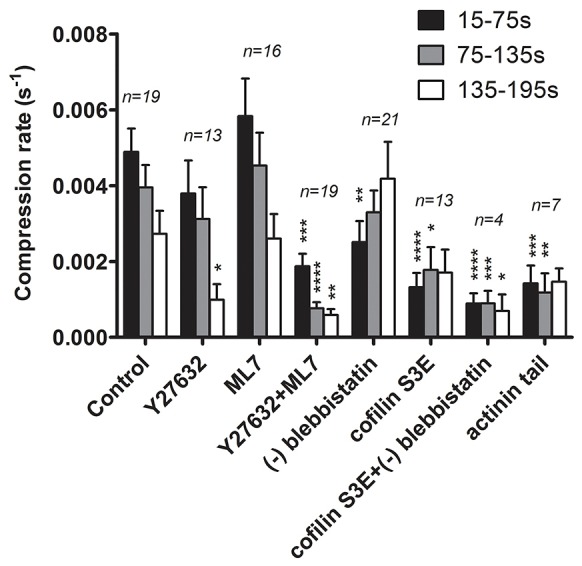
**Vesicle compression rates after genetic or pharmacological inhibition of proteins participating in actin coat compression.** Compression rates were calculated for 60-s intervals during vesicle compression as described in the Materials and Methods section. Compression rates are shown as mean±s.e.m. and the numbers indicate the number of vesicles. *P<0.05, **P<0.01; ***P<0.001, ****P<0.0001.

**Fig. 7.**
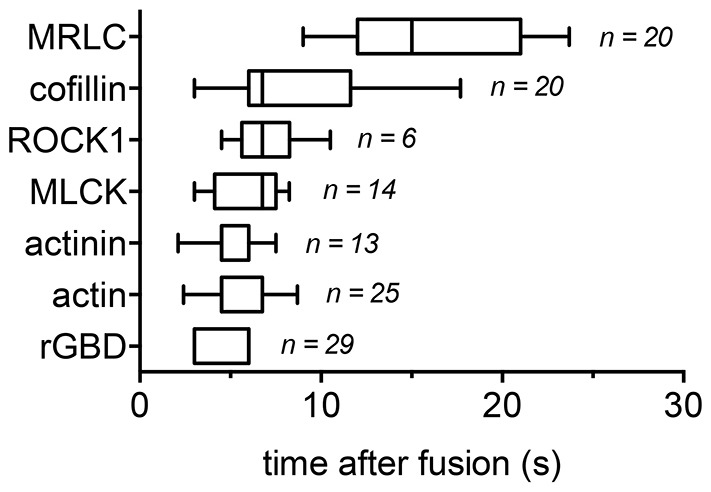
**Chronology of protein recruitment to the lamellar body membrane after fusion.** The time of fluorescence intensity increase was measured for fluorescently labelled proteins in the region of interest around the fusing lamellar body with respect to fusion start. The time point of fusion was determined by decrease in LysoTracker fluorescence. (n indicates the number of fused vesicles). The box represents the 25–75th percentiles, and the median is indicated. The whiskers show the 10–90th percentiles.

In contrast to the moderate effect of expressing constitutively active cofilin, expression of an inactive phospho-cofilin mimetic (cofilin S3E) had a very strong and significant inhibitory impact on actin coat contraction (Fig. 4E; Fig. 6, *P*<0.01 for 45–195 s). This, at first glance contradictory result, suggests that regulated actin depolymerisation is essential for coat contraction. However, it has recently been proposed that regulated actin depolymerisation mediated by cofilin can produce substantial contractile forces (Mendes Pinto et al., 2012; Mseka and Cramer, 2011; Sun et al., 2010). In such a model, actin depolymerisation requires the presence of actin crosslinkers to drive sliding between the actin filaments and produces the force needed for compression (Mendes Pinto et al., 2012). In line with such a model, immunofluorescence staining confirmed that α-actinin was localised on actin coats around fused vesicles (Fig. 5A), and live-cell experiments showed that actinin–GFP was recruited to fused vesicles immediately after fusion (4.8±0.5 s, *n* = 13, Fig. 7; Fig. 5B; mean±s.e.m.). α-actinin consists of an actin-binding head domain and a tail domain, which enables dimerisation of α-actinin in anti-parallel orientation to promote crosslinking of adjacent actin filaments. Overexpressing either actinin head or tail domains, which occupy actin-binding sites or interfere with dimerisation of wild-type actinin, respectively, had a strong and significant inhibitory effect on actin coat contraction (Fig. 5C). The compression rates were reduced throughout coat contraction, with the strongest impact on early stages of contraction (*P* = 0.0002 for 15–75 s, *P* = 0.002 for 75–135 s and *P* = 0.08 for 135–195 s; Fig. 6). These data strongly suggest that regulated actin depolymerisation and crosslinking is essential for coat contraction. The myosin II inhibitor (−)-blebbistatin slightly reduced actin coat compression rates in cofilin-S3E-transfected cells; however, the reduction was not significantly different from the effect of cofilin S3E alone (*P* = 0.18 at time = 165 s; Fig. 4E; Fig. 6). The importance of actin depolymerisation for vesicle compression is further supported by our observation that pharmacological inhibition of actin depolymerisation by 1 µM jasplakinolide resulted in significantly reduced coat contraction (supplementary material Fig. S3A,B; *P* = 0.004–0.04 for time = 45–165 s).

In contrast, actin polymerisation did not appear to play a role in actin coat compression. Treatment of ATII cells with latrunculin B or cytochalasin D inhibited actin coat formation on fused vesicles (P.M., unpublished observation; Miklavc et al., 2012). However, when latrunculin B or cytochalasin D were added 2 min after stimulation of lamellar body exocytosis actin coats still formed on lamellar bodies fusing within the initial 2 min after stimulation. Under these conditions, latrunculin B or cytochalasin D did not result in coat disintegration but also did not affect coat contraction (supplementary material Fig. S3C–F). Although these experiments cannot fully exclude the possibility that initial actin polymerisation on fused lamellar bodies contributes to vesicle compression, these observation suggests that actin filaments, once formed, remain on the fused vesicle until compression is complete and that continuous actin polymerisation is not likely to be a prerequisite and driving force for actin coat compression.

In summary, our data provide strong evidence that actin coat formation and contraction on fused lamellar bodies is a tightly regulated process essential for efficient secretion of pulmonary surfactant. We propose a model, whereby immediately after lamellar body fusion with the plasma membrane Rho proteins are recruited to the fused lamellar body to initiate actin polymerisation (Miklavc et al., 2012) and activate ROCK. Alpha-actinin is recruited to the actin coat to stabilise and crosslink newly formed filaments. Subsequent translocation of ROCK1 and MLCK1 activates myosin II and regulates actin severing activity of cofilin on actin coats. Actin depolymerisation/crosslinking and myosin II then join forces to contract actin coats around fused secretory vesicles and drive secretion.

## DISCUSSION

We have recently reported that actin coating of fused vesicles and coat contraction are necessary for efficient surfactant extrusion from lamellar bodies in ATII cells. We have also demonstrated a role for the actomyosin complex in actin coat compression (Miklavc et al., 2012). However, detailed molecular mechanisms regulating actin coat contraction were still missing. In this study, we now provide detailed kinetics information on the molecules regulating actin coat contraction. Understanding the sequence of molecule translocation and activation, and hence the players present at a time, is essential for unravelling mechanistic aspects of coat contraction.

Our data clearly demonstrate that myosin II activity is not the main driving force for actin coat contraction but rather seems to have a facilitating function. Inhibition of myosin II activity decreased vesicle compression rates, but did not prevent coat contraction. This is in line with observations from most secretory systems where actin coating has been reported. In all cases inhibition of myosin II activity did not completely block actin coat contraction (Jerdeva et al., 2005; Masedunskas et al., 2011; Miklavc et al., 2012; Nemoto et al., 2004; Nightingale et al., 2011; Yu and Bement, 2007b). Hence, the question remains as to what is the role for myosin during coat contraction and what other mechanisms might contribute to compression.

Our data suggest that regulated actin depolymerisation and actin fragment crosslinking drive coat contraction. In particular, our results highlight an important role for cofilin and α-actinin in actin coat compression and indicate that a compression model initially proposed for cytokinetic rings (Mendes Pinto et al., 2012; Sun et al., 2010) might also apply for actin coat compression in ATII cells. In such a model, actin depolymerisation in the presence of crosslinkers produces filament sliding. Specifically, when a cut in a filament occurs near a filament crosslinking site, thermal fluctuation of the crosslinker allows its reattachment with the new filament end, leading to sliding of the filament caused by elastic energy stored in an elongated crosslinker. In such a model, contraction is independent of motor activity and is also independent of actin filament organisation (Mendes Pinto et al., 2013; Zumdieck et al., 2007), two conditions likely to be the case in actin coating of fused lamellar bodies. We already know that contraction proceeds even when myosin II motor activity is inhibited. Owing to the spherical alignment of the actin coat, it is easily conceivable that actin filaments are orientated rather isotropically and not strictly in antiparallel arrays. Hence, relying on a ‘sliding filament’ mechanism in which bipolar myosin filaments walk along antiparallel actin filaments might not be sufficient for efficient and complete contraction of the actin coat. Such a structural architecture of the coat would also suggest that myosin might have alternative functions other than direct force generation. The main function of myosin II might not be active filament sliding and coat contraction, but rather organising or stabilising the actin coat. Such a model is supported by observations in pancreatic and parotid acinar cells where myosin II and actin coats stabilise fused granules rather than provide a contraction force (Bhat and Thorn, 2009; Larina et al., 2007; Nemoto et al., 2004; Segawa and Yamashina, 1989). Moreover, myosins have been shown to play a role in regulating or modulating actin coat symmetry (Yu and Bement, 2007b), and myosin and cofilin binding to actin are mutually exclusive (Galkin et al., 2011). Our observation that (over)expression of MRLC(wt)–GFP also had a slightly inhibitory effect on the vesicle compression rate compared to untreated cells (see Fig. 1C), supports such a model and suggests that increased crosslinking of actin filaments with myosin II might inhibit efficient vesicle compression. Further evidence for a minor role for myosin II in direct force generation also comes from the observation that recruitment of myosin to fused vesicles is slow and hence might not be suited to driving efficient content expulsion, in particular during the initial phase of coat contraction. Recruitment of myosin II is significantly delayed compared to actin coat formation and recruitment of actin depolymerising and crosslinking molecules following lamellar body exocytosis. In addition, in *Xenopus* oocytes myosin II recruitment to fused vesicles is only observed following initiation of coat compression (Yu and Bement, 2007b). Slow translocation of myosin could also support a dual role for myosin. Initially, at low concentrations myosin might serve an organising role and at late stages, with high concentrations, could also contribute to actin depolymerisation contributing to the depolymerisation- and crosslinking-derived force generation (Guha et al., 2005; Reymann et al., 2012). From our data we cannot finally assess which is the predominant role for myosin in coat contraction, yet it is likely that myosin contributes to force generation (probably at late stages of compression) as well as actin filament organisation.

Irrespective of the role of myosin for coat compression and secretion, our data clearly support a role for regulated actin depolymerisation and actin fragment crosslinking for coat contraction. In line with previous results where we demonstrated that Rho activation is necessary for actin coat formation (Miklavc et al., 2012), we report here that Rho GTPases are also responsible for regulating coat compression through the activation of ROCK1. Previous results have suggested that RhoA is responsible for actin coat formation, but could not exclude that Rho proteins other than RhoA might be involved (Miklavc et al., 2012). Here, we provide additional evidence that RhoA, but not RhoB or RhoC, is likely to be the main isoform involved in coat formation. However, we cannot yet fully exclude that RhoB, which also translocates to fused lamellar bodies, also plays some role. RhoB has been found to also induce actin formation through formins (Fernandez-Borja et al., 2005; Wallar et al., 2007). With regards to regulation of contraction, the picture again is not univocal. Expression of dnRhoA as well as dnRhoB significantly slowed down actin coat contraction. Both are present on fused lamellar bodies and both can signal to ROCK1 and MLCK (Ridley, 2006). Hence it is possible that either of the two isoforms contributes to regulating coat contraction. It is still unclear what the specific mechanisms that lead to recruitment of RhoA and RhoB are. It has been shown that RhoA and RhoB can be activated by the same guanine-nucleotide-exchange factor, but alternatively, RhoB, which is predominantly localised to the plasma membrane (Ridley, 2006) could simply diffuse into fused lamellar body membranes upon membrane mixing (Sokac and Bement, 2006). Separate recruiting mechanisms (and kinetics) could potentially account for the differences in function, with RhoA promoting coat formation and contraction and RhoB mainly acting on contraction. Further experiments are warranted to uncover the detailed mechanisms for specificity of selective Rho GTPase recruitment to fully understand the individual contributions of individual Rho GTPases to actin coat formation and contraction.

It is interesting that the signalling cascades and contractile force-generating mechanisms leading to actin assembly and contractility of vesicle coats in ATII cells closely resemble signalling pathways involved in actin remodelling during cytokinesis (Castrillon and Wasserman, 1994; Watanabe et al., 2008), cell motility (Mseka and Cramer, 2011) and β2 integrin (CR3)-mediated phagocytosis (Caron and Hall, 1998; Colucci-Guyon et al., 2005; Olazabal et al., 2002). In all of these processes, Rho-dependent actin polymerisation is conducted by formins (Castrillon and Wasserman, 1994; Miklavc et al., 2012; Watanabe et al., 2008), whereas contractility is mediated through ROCK-dependent activation of myosin II (Araki et al., 2003; Matsumura et al., 2011; Olazabal et al., 2002; Reichl et al., 2008) and/or cofilin inhibition (Deschamps et al., 2013; Matsui et al., 2002). Considering that bundling, depolymerisation and crosslinking of cytoskeletal filaments has been found in processes as different as cytokinesis, cell migration and phagocytosis (Deschamps et al., 2013), it is likely that these types of filament rearrangements form a well conserved and general mechanism for force generation (Sun et al., 2010). Hence, it is probably not surprising that bundling, depolymerisation and crosslinking are also found in secretory systems where granule compression is required for efficient extrusion of poorly soluble material.

To our knowledge, this is the first study to demonstrate an additional actin coat compression mechanism for the post-fusion phase of exocytosis that is complementary to myosin-mediated contraction. It is tempting to speculate whether cofilin- and actinin-driven coat compression is also found in other secretory cell types, where actin coating of granules has already been reported. Some findings support a more widespread role for such a mechanism. Cofilin has been found to play a role in exocytosis of insulin-containing granules in β-cells (Uenishi et al., 2013) and in secretory granule exocytosis in adrenal chromaffin cells (Birkenfeld et al., 2001). However, in these studies a specific role during the post-fusion phase was not investigated and it was proposed that cofilin-mediated actin depolymerisation regulates the actin network in the pre-fusion stage of exocytosis, enabling vesicle fusion with the plasma membrane. Similarly, α-actinin has been found on the membrane of chromaffin granules (Jockusch et al., 1977) although its precise function is still unknown. A recent histological study of salivary gland cells [where actin coats have been well studied (Masedunskas et al., 2011)], reported that there was localisation of cofilin to the apical cell membrane (Stoeckelhuber et al., 2012). Hence, it is tempting to speculate, that cofilin-mediated actin depolymerisation and actin crosslinking plays a role in compression of actin coats in secretory cell types other than type II cells.

We can only speculate what the purpose of such an elaborate and complex secretion mechanism is. It is feasible that complementary force generation by several mechanisms is necessary to even generate the required force for secretion. In particular in the case of large macromolecular vesicle cargoes, fusion pores are substantial barriers to content release (Miklavc et al., 2011; Neuland et al., 2014) and a large amount of force is required for secretion. It has been demonstrated that surfactant is ‘squeezed’ through narrow fusion pores (Haller et al., 2001) and that this leads to considerable transformation of the macromolecular structure of the tightly packed lipid layers (Goerke, 1998). Therefore additive force generation by myosin-driven filament sliding and actin depolymerisation and crosslinking might be required to provide sufficient force for vesicle compression.

Alternatively, it is also possible that the regulated sequential recruitment of molecules results in coordinated contraction of the secretory vesicles. It has been demonstrated in other systems that coordinated initiation and progression of coat contraction is essential for secretion (Masedunskas et al., 2011; Nightingale et al., 2011; Yu and Bement, 2007b). In the case of Weibel-Palade bodies, an actin filament ring initiates distally from the fusion pore on the bottom of an open granule and, travelling along the fused granule, acts as a minicytokinetic ring to exert force, pushing von Willebrand factor out on the other end into the extracellular environment (Babich et al., 2008). We cannot fully resolve the detailed spatial kinetics of molecule recruitment to fused vesicles (i.e. lateral diffusion from the plasma membrane or direct recruitment from cytoplasm). However, it is conceivable that force generation on fused lamellar bodies needs to be orchestrated to result in polarised directed force generation facilitating outwards-directed expulsion (squeezing) of the lipidic cargo. This might be crucial for secretion-dependent transformation (and activation) of surfactant (Singer et al., 2003) or to prevent premature closure of the fusion pore.

**Fig. 8.**
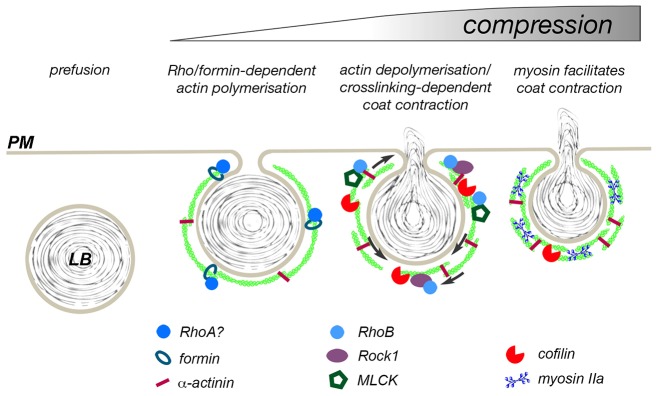
**Proposed model for actin coat contraction.** Upon fusion of the lamellar body (LB) with the plasma membrane (PM), Rho GTPases (possibly RhoA) are recruited to the fused lamellar body and initiate formin-dependent actin polymerisation on the fused vesicle. Actin filaments are crosslinked through α-actinin. Subsequently RhoB, ROCK1, MLCK and cofilin are recruited to actin-coated lamellar bodies. Cofilin-dependent depolymerisation of actin filaments and crosslinking of new filament ends by α-actinin results in contraction of the actin coat (arrows) in a manner similar to a recently proposed mechanism for cytokinetic ring contraction (Mendes Pinto et al., 2012). Cofilin activity is regulated by ROCK1, inhibiting complete and premature disintegration of the coat. In addition, myosin II is recruited to actin coats and facilitates coat contraction in particular during late stages of compression.

In summary, our data suggest that actin coat contraction is a highly regulated process and that selective and sequential recruitment of molecules regulating coat contraction is essential for efficient vesicle compression. Based on our previous findings and data from this study we propose a model whereby shortly after fusion of a lamellar body with the plasma membrane Rho GTPases are selectively recruited to the fused vesicle. Active RhoGTPases initiate polymerisation of actin fibres, probably through formins, and act as master regulators for coat contraction, orchestrating controlled actin depolymerisation and myosin recruitment and activation (Fig. 8). Whether myosin is essential for force production or rather organises actin filaments is yet to be determined. Overall, myosin-II- and cofilin-mediated actin depolymerisation and subsequent crosslinking of actin fibres with α-actinin leads to force generation essential for actin coat compression and surfactant extrusion from secretory vesicles in ATII cells.

## MATERIALS AND METHODS

### Antibodies

Antibodies against ROCK1 (monoclonal), ROCK2 (polyclonal), α-actinin (polyclonal) and ABCa3 (P180 lamellar body protein, monoclonal) were from Abcam (Cambridge, UK). Fluorescently labelled secondary antibodies were obtained from Molecular Probes (Life Technologies, Karlsruhe, Germany).

### Plasmids and adenoviruses

Plasmids expressing MRLC(wt)–GFP, MRLC2(AA)–GFP and MRLC2(DD)–GFP were a kind gift from Hiroshi Hosoya (Hiroshima University, Hiroshima, Japan) (Iwasaki et al., 2001); ROCK1–YFP was generously provided by Garreth Jones (King's College London, London, UK) (Shea et al., 2008), wt- and dn-MLCK-GFP were a generous gift from Anne Bresnick (Albert Einstein College of Medicine, New York, NY) (Dulyaninova et al., 2004), and cofilin wt, S3A and S3E constructs linked to YFP or DsRed were kindly provided by Kensaku Mizuno (Tohoku University, Sendai, Japan) (Kaji et al., 2008). GFP–rGBD and GFP–α-actinin were purchased from Addgene (ID 26732 and 11908, respectively). Adenoviruses expressing GFP–rGBD were produced using the Adeno-ONE cloning and expression kit (Sirion Biotech, Martinsried, Germany). Briefly, rGBD–GFP was cloned into shuttle vector pO6A5 and transformed into *E. coli* (BA5-FRT). Transformation was followed by flp-mediated recombination of pO6A5 and SIR-BAC-Ad5 in bacteria. Purified BAC-DNA was digested with *Pac*I and used for transfection of HEK 293 cells with jetPEI transfection reagent (Polyplus transfection, Illkirch, France). Adenoviral particles were isolated using the ViraBind adenovirus purification kit (Cell Biolabs, San Diego, USA). Constructs expressing α-actinin head and tail were generated by PCR-amplifying amino acids 1–247 and 249–892 (inserting a start codon upstream of amino acid 249) from human α-actinin, respectively, and cloning the products into pEGFP-N1 using *Eco*RI and *Hin*dIII restriction sites (Clontech, TakaraBio, France).

Dominant negative (T19N) RhoA, RhoB and RhoC isoforms were obtained from the Missouri S&T cDNA Resource Center (Rolla, USA) and cloned in multiple cloning site A of the pIRES vector (Clontech, Mountain View, USA) using the restriction sites *Xho*I and *Eco*RI (RhoA T19N), *Nhe*I and *Eco*RI (RhoB T19N), and *NheI* and *XhoI* (RhoC T19N). Fluorescent protein YFP was inserted into multiple cloning site B of the pIRES vector using the *Sal*I and *Not*I restriction sites. GFP-tagged RhoA, RhoB and RhoC constructs were purchased from Addgene (Plasmid IDs: 23224, 23225, and 23226, respectively). Cells were transfected using the Nucleofector 4D system (Lonza, Germany).

Adenoviruses expressing actin–GFP, actin–DsRed and lyn–DsRed were as recently described (Miklavc et al., 2012; Miklavc et al., 2009).

### Cell isolation

ATII cells were isolated from Sprague-Dawley rats according to the procedure of Dobbs et al. (Dobbs et al., 1986) with minor modifications as recently described (Miklavc et al., 2010; Thompson et al., 2013). After isolation, cells were seeded on glass coverslips, cultured in MucilAir (Epithelix, Switzerland), and used for experiments for up to 48 h after isolation. All animal experiments were performed according to approved guidelines.

### Experimental conditions

Experiments were performed as recently described (Miklavc et al., 2010). For all experiments cells were kept in bath solution (in mM: 140 NaCl, 5 KCl, 1 MgCl_2_, 2 CaCl_2_, 5 glucose, 10 Hepes, pH 7.4). ATII cells were stimulated with 100 µM ATP (Sigma, Schnelldorf, Germany). Cells were incubated with inhibitors for Rho kinase (Y27632, 10 µM, overnight), myosin light chain kinase (ML-7, 30 µM, 20 min) and myosin [(−)-blebbistatin, 25–50 µM] overnight, for 20 min or for 2 h, respectively. Y27632 was purchased from Sigma, ML7 and (−)-blebbistatin from Calbiochem (Darmstadt, Germany). All fluorescent dyes were purchased from Molecular Probes (Invitrogen, Karlsruhe, Germany).

### Semi-quantitative RT-PCR

Total RNA was isolated from 10^6^ ATII cells directly after isolation or following 48 h of culture in MucilAir medium with an RNeasy MiniKit (Qiagen, Hilden, Germany). Reverse transcription was performed on 0.8 µg to 1.3 µg total RNA using the SuperScript VILO cDNA synthesis kit according to manufacturer's protocol and validated QuantiTect primer assays (Qiagen, Hilden Germany) Amplification was performed on a realplex2 mastercycler (Eppendorf, Hamburg, Germany) using the XPress Syber Green ER qRT-PCR super mix. Each reaction was carried out on cDNA from ≥three independent isolations (cDNAs were used at 1-, 10- and 100-fold dilutions). Specificity of PCR reactions was confirmed by melting points analysis of PCR products. Realplex software (Eppendorf, Hamburg, Germany) was used for data acquisition and analysis. Correction for PCR performance as well as quantification relative to housekeeping gene *Hmbs* was carried out as described previously (Pfaffl, 2001).

### Immunofluorescence

For immunofluorescence staining, cells were washed twice in DPBS (pH 7.4, Biochrom, Berlin, Germany), fixed for 20 min in 4% paraformaldehyde (Sigma, Schnelldorf, Germany) in DPBS and permeabilised for 10 min with 0.2% saponin and 10% FBS (Thermo Scientific, Bonn, Germany) in DPBS. Cells were subsequently stained with primary (1∶300) and secondary (1∶400) antibodies in PBS, 0.2% saponin and 10% FBS. Images were taken on an inverted confocal microscope (Leica TCS SP5, Leica, Germany) using a 63× lens (Leica HCX PL APO lambda blue 63.0×1.40 OIL UV). Images for the blue (DAPI), green (Alexa Fluor 488), red (Alexa Fluor 568) and far red (Alexa Fluor 647) channels were taken in sequential mode using appropriate excitation and emission settings.

### Fluorescence imaging

Fluorescence imaging experiments were performed on an iMic digital microscope (Till Photonics, Germany) and on a Cell Observer inverse microscope (Zeiss, Germany). Before experiments, cells were incubated with LysoTracker Red or LysoTracker Blue (LTR or LTB, LifeTechnologies, Germany; 10–100 nM, 10–20 min) to detect lamellar body fusions. LysoTracker dyes accumulate in lamellar bodies and rapidly diffuse out of the vesicle after fusion (Haller et al., 1998). Images were acquired at a rate of 0.3–0.6 Hz using iMic Online Analysis (Till Photonics, Germany) or MetaFluor (Molecular Devices, Ismaning, Germany) software, respectively, and using a 488 nm excitation filter for GFP and 568 nm excitation filter for LTR.

### Image analysis and data presentation

Images were analysed using iMic Online Analysis (Till Photonics, Germany), MetaFluor Analyst (Molecular Devices, Ismaning, Germany) and Fiji (NIH, Bethesda, United States). MS Excel and GraphPad Prism 5 were used for statistics, curve fitting and graph design. Unless otherwise stated all data are presented as mean±s.e.m.

Actin coat contraction was analysed by measuring the perimeter of individual actin rings at indicated time-points after fusion. For determining the onset of lamellar body fusion, LTR fluorescence was analysed in a region encircling the fusing lamellar body (Miklavc et al., 2014; Miklavc et al., 2012; Miklavc et al., 2011). Actin coat compression rate was calculated for three different 60 s time intervals of actin coat compression (15–75 s, 75–135 s and 135–195 s after fusion) using equation for ring compression from Mendes-Pinto et al. (Mendes Pinto et al., 2012): 1/D(dD/dt), where D denotes ring diameter.

## Supplementary Material

Supplementary Material
