# Supplementary material for: Actin depolymerisation and crosslinking join forces with myosin II to contract actin coats on fused secretory vesicles
Source: J Cell Sci. 2015 Mar 15;128(6):1193–203. doi: 10.1242/jcs.165571 (PMC4359923; doi:10.1242/jcs.165571)
Supplement: Supplementary Material [file supp_128_6_1193__index.html]

Actin depolymerisation and crosslinking join forces with myosin II to contract actin coats on fused secretory vesicles — Supplementary Material 

# Actin depolymerisation and crosslinking join forces with myosin II to contract actin coats on fused secretory vesicles

## JCS165571 Supplementary Material

**Files in this Data Supplement:**

- **Supplementary Material**
