# Supplementary material for: Actin depolymerisation and crosslinking join forces with myosin II to contract actin coats on fused secretory vesicles
Source: J Cell Sci. 2015 Mar 15;128(6):1193–203. doi: 10.1242/jcs.165571 (PMC4359923; doi:10.1242/jcs.165571)
Supplement: Supplementary Material [file supp_128.6.1193_JCS165571.pdf]

Miklavc et al. Supplementary Information:

Figure S1

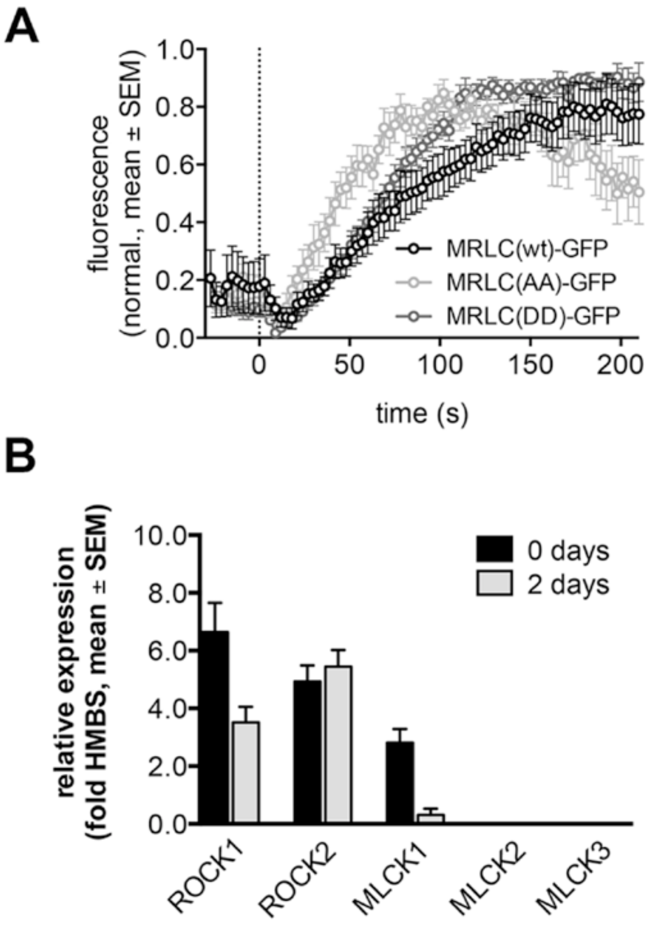

Supplementary Figure S1:

Translocation of different MRLC constructs and expression of Rock and MLCK isoforms

A) GFP-labelled AA and DD mutants of MRLC translocated to the LB membrane with similar kinetics as wt MRLC-GFP. Translocation was measured as GFP fluorescence increase on the region of interest around a fusing LB. Time point of fusion (dashed line) was determined by decrease in LysoTracker fluorescence.

B) RT-PCR showed that both isoforms of Rock were expressed, whereas only MLCK1 was expressed.in isolated ATII cells.

Figure S2

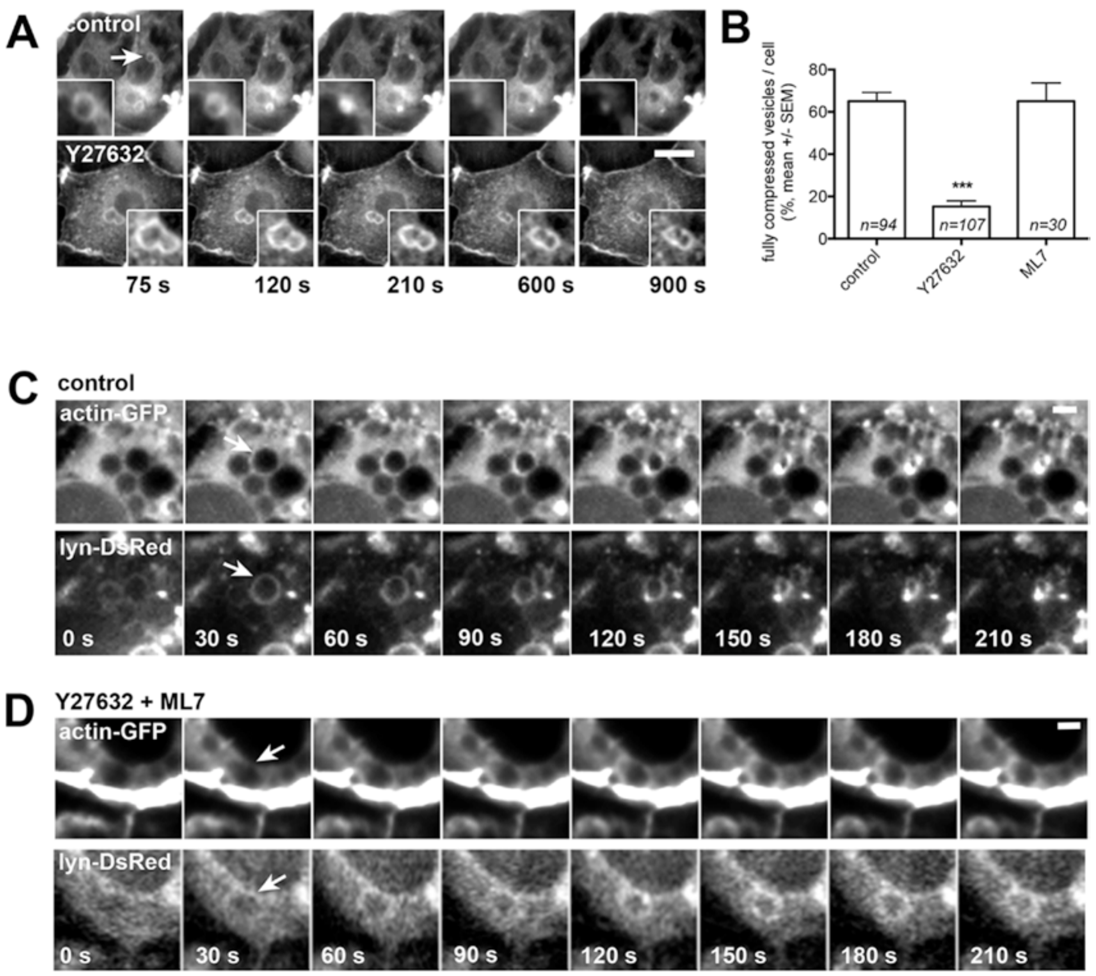

Supplementary Figure S2:

Inhibition of Rock by Y27632 inhibitor prevented completion of actin coat compression.

A) Actin-GFP fluorescence images of a control cell showing full compression of actin coats during 15 min image acquisition time (arrows, upper row), and only partial compression in a cell after Y27632 treatment (arrow, bottom row). Numbers indicate time after stimulating the cells for fusion with ATP, inserts show the enlarged view of the fusing vesicles. Scale bar = 10 µm.

B) The proportion of actin coats with complete compression was significantly reduced after treatment with Y27632 ($p<0.0001$, n=112). ML7 inhibitor did not inhibit complete compression (n=30 and 94 for ML7 and control, respectively). The percentage of full compressions was calculated for ATII cells with at least one vesicle fusion in the first 5 min after ATP stimulation.

C) + D) Simultaneous imaging of actin-GFP (upper row) and membrane marker lyn-DsRed (lower row). Lyn-dsRed rapidly diffuses into the LB membrane after fusion (arrow). Compression of lyn-dsRed labelled LB and contraction of actin coat correlate under control conditions (C). Inhibition of Rock1 and MLCK1 (Y27632 + ML7) inhibits actin coat contraction and stalls compression of fused LBs (D). Time stamps indicate time after fusion. Scale bar: 2 μm

Figure S3

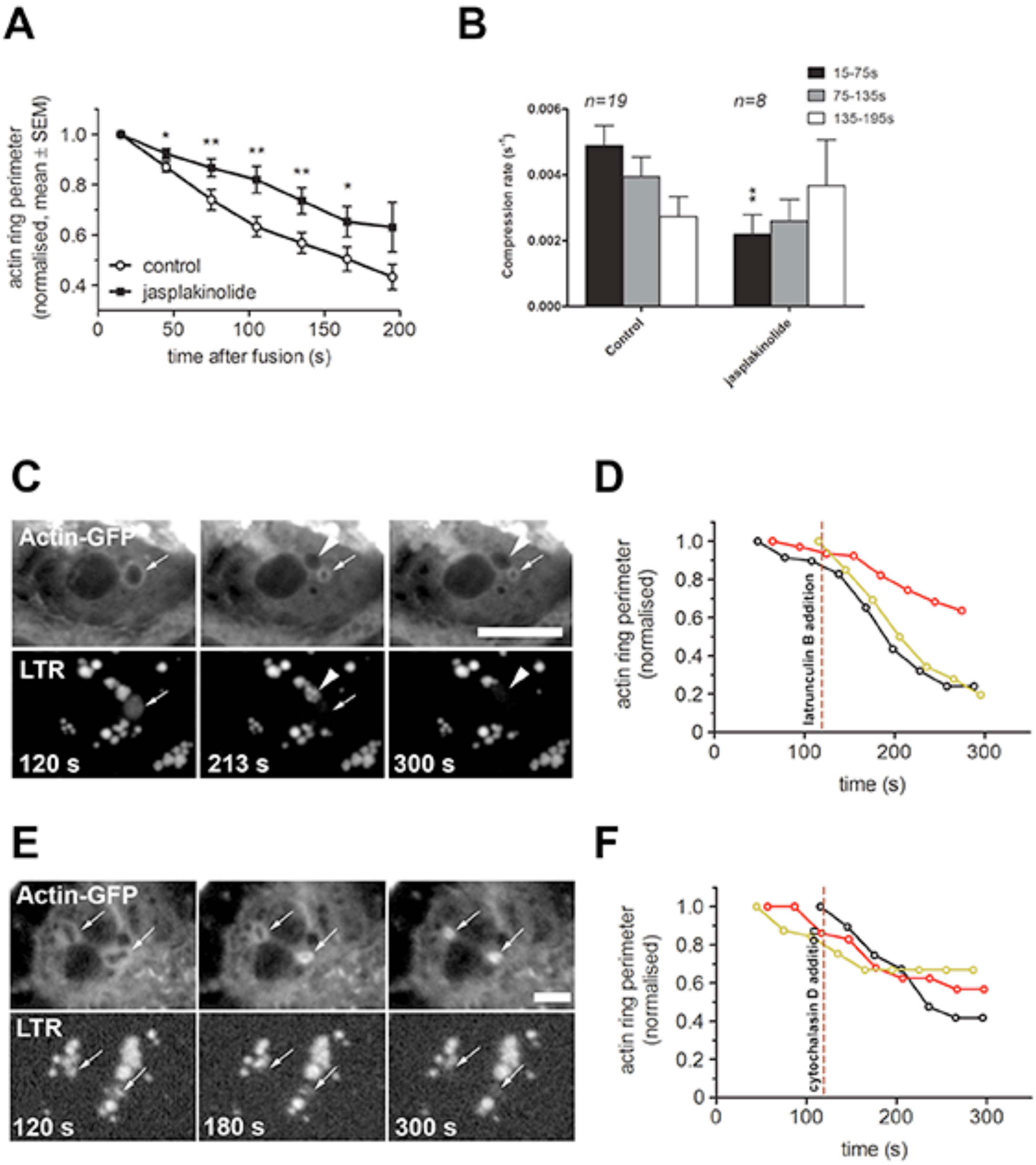

Supplementary Figure S3:

Effect of jasplakinolide, latrunculin B, and cytochalasin D on compression of actin coats.

A) Treatment with jasplakinolide (1µM, 10 min) significantly reduced vesicle compression compared to control cells (n = 19 and 8 for control and jasplakinolide, respectively).

B) Vesicle compression rates were significantly reduced in cells treated with 1 µM jasplakinolide for 10 min compared to control cells ($p<0.05$ for times 45-165s; n=19 and 8 for control and jasplakinolide, respectively).

C) Latrunculin B (10 µM) was added to actin-GFP transfected cells 120 s after stimulating the cells for fusion when some actin coats were already formed around fused vesicles (arrow, upper row). Vesicles fusing after latrunculin B addition (arrowhead; t=213s) did not acquire actin coats, whereas already formed actin coats persisted until the end of experiment (t=300s). Upper row: actin GFP fluorescence, bottom row: LysoTracker fluorescence. Note the decrease in LysoTracker fluorescence after fusion. Scale bar = 10 µm.

D) Compression rates of single vesicles which fused and acquired actin coats before addition of latrunculin B (time point of latrunculin addition is indicated with dashed line). Note that the vesicles continued to compress after latrunculin B addition.

E) Cytochalasin D (10 µM) was added to ATII cells expressing actin-GFP 120 s after ATP stimulation. Under these conditions actin coats still formed on fused vesicles before cytochalasin D addition (arrows, upper row). Already formed actin coats persisted until the end of experiment (t=300s). Upper row: actin GFP fluorescence, bottom row: LysoTracker red fluorescence. Note the decrease in LysoTracker fluorescence after fusion. Scale bar = 5 µm.

F) Compression rates of single vesicles which fused and acquired actin coats before addition of cytochalasin D (time point of cytochalasin addition is indicated with dashed line). Note that the vesicles continued to compress after cytochalasin addition.
